# The Potential of Ladle Slag and Electric Arc Furnace Slag use in Synthesizing Alkali Activated Materials; the Influence of Curing on Mechanical Properties

**DOI:** 10.3390/ma12071173

**Published:** 2019-04-10

**Authors:** Mark Češnovar, Katja Traven, Barbara Horvat, Vilma Ducman

**Affiliations:** 1 Slovenian National Building and Civil Engineering Institute, Dimičeva 12, 1000 Ljubljana, Slovenia; mark.cesnovar@zag.si (M.Č.); katja.traven@zag.si (K.T.); barbara.horvat@zag.si (B.H.); 2 Jozef Stefan International Postgraduate School, Jamova cesta 39, 1000 Ljubljana, Slovenia

**Keywords:** alkali activation, ladle slag, electric arc furnace slag, ageing, curing conditions

## Abstract

Alkali activation is studied as a potential technology to produce a group of high performance building materials from industrial residues such as metallurgical slag. Namely, slags containing aluminate and silicate form a useful solid material when activated by an alkaline solution. The alkali-activated (AA) slag-based materials are promising alternative products for civil engineering sector and industrial purposes. In the present study the locally available electric arc furnace steel slag (Slag A) and the ladle furnace basic slag (Slag R) from different metallurgical industries in Slovenia were selected for alkali activation because of promising amorphous Al/Si rich content. Different mixtures of selected precursors were prepared in the Slag A/Slag R ratios 1/0, 3/1, 1/1, 1/3 and 0/1 and further activated with potassium silicate using an activator to slag ratio of 1:2 in order to select the optimal composition with respect to their mechanical properties. Bending strength of investigated samples ranged between 4 and 18 MPa, whereas compressive strength varied between 30 and 60 MPa. The optimal mixture (Slag A/Slag R = 1/1) was further used to study strength development under the influence of different curing temperatures at room temperature (R.T.), and in a heat-chamber at 50, 70 and 90 °C, and the effects of curing time for 1, 3, 7 and 28 days was furthermore studied. The influence of curing time at room temperature on the mechanical strength at an early age was found to be nearly linear. Further, it was shown that specimens cured at 70 °C for 3 days attained almost identical (bending/compressive) strength to those cured at room temperature for 28 days. Additionally, microstructure evaluation of input materials and samples cured under different conditions was performed by means of XRD, FTIR, SEM and mercury intrusion porosimetry (MIP).

## 1. Introduction

Alkali activation technology offers the possibility to utilize large amounts of aluminosilicate-rich secondary products such as fly ash from thermoplants [1,2,3], slag from metallurgical processes [4,5] and waste glass [6] for a useful new group of building products. Steel slag-based alkali activated materials (AAM), have a high mechanical strength, show good fire resistance and high thermal resistance at elevated temperatures, and, in the case of low density, they further exhibit low thermal conductivity [7]. As reported, various types of slag can be alkali activated, either as a single precursor or mixed with other aluminosilicates (e.g., fly or bottom ash, metakaolin, waste glass and ceramics) [8,9,10].

The slags are residues which are produced during the high temperature separation of metallic and non-metallic materials in metallurgical processes [8]. Due to their mineral composition these industrial by-products should be recycled and reused, not only for the purpose of reducing waste in the environment, but also for the economic recovery of the original materials. The blast furnace (BF) iron slag, also known as a ground granulated blast furnace slag (GGBFS), the electric arc furnace carbon or stainless steel slag (EAF-C/S), and the ladle furnace basic slag (LS), also called the white slag, have been regarded as precursors which exhibit good mechanical properties following alkali activation and can be used to create valuable materials for building and civil engineering works [9,10,11,12,13]. Electric arc furnace slag (EAFS) was investigated as the potential partial substitute for cement in mortar. Mohmoud et al. presented that EAFS when replaced by 15 to 30% with cement decreased compressive strength in intial stages (first 14 days), but contributed to higher strength after 28 days compared to mortars without EAFS addition [14]. Steel slags can also be employed as an alternative to fly ash which is already well known cement substituent due to its pozzolanic nature [15]. Steel slag can also be utilized in many different areas, such as an adsorbent for heavy metals, as a sintering additive, in asphaltic mixes, and for the production of cement and concrete as a raw material for the Portland clinker fabrication [16,17,18]. 

Alkali activators commonly used in the process of AAM are sodium silicate (Na_2_SiO_3_), potassium silicate (K_2_SiO_3_) and sodium or potassium hydroxide solution (NaOH, KOH). K^+^ is more basic than Na^+^, thereby allowing a higher solubility and dissolution of aluminosilicates, which consequently leads to improvements in polymerization and hardening of the AAM [19]. Activators can be used separately or as mixtures, which has been reported to enhance the mechanical properties of AAM [10,20]. In most cases the main reactive product of activated slag mortars with an activator such as NaOH or KOH is a highly amorphous sodium alumino-silicate hydrate (N-A-S-H) and calcium alumino-silicate hydrate (C-A-S-H) gel [21]. Also C-S-H gel, which is a common product from the hydration of Portland cement, is present in slag based AAM with low Ca/Si ratios as suggested by Yip et al. [22]. 

Besides the reactivity potential of the slag with an alkali activator, which depends on the chemical composition of the slag and the amount of aluminosilicate amorphous phase, surface morphology and particle size distribution, the addition of an alkaline solution and ageing and curing conditions should be taken into account. The development of strength in slag-based AAMs with respect to curing regimes has been studied and presented in various publications [4,19,23,24,25]. Curing at elevated temperatures can greatly affect the chemical reaction with the gel formation and geopolymerization. Researches determined that curing temperatures from 50–80 °C are sufficient for successful geopolymer hydratation. Rapid set of geopolymerization in the first 2 to 5 h, when curing takes place, results in the significant percentage of total compressive strength, the strength increase beyond 48 h of curing is not significant [19,24]. After that, ageing takes place. Nasr et al. investigated the effect of ambient curing of the ground granulated blast furnace slag activated with Na_2_SiO_3_ at 23 °C with 33% humidity for 28 days, the effect of water curing, where samples were demolded after one day and immersed in water tank at 25 °C for 28 days, the effect of hydrothermal curing at 135 °C, 2.3 bar of pressure for 2 h after demolding for 28 days. The influence of heating at elevated temperatures was also studied. As seen in the report, when the temperature is increased for the specimens cured at room temperature the mechanical strength decreased. That’s not the case for water and hydrothermal cured specimens where increase in temperature has a positive effect on the mechanical strength. It was concluded that temperature accelerates the hydratation process like for C-S-H formation and more precursor particle are activated. Water absorption is greater in the case of water type curing prior temperature exposure, as authors suggests that is due to the large pore formation resulting from accelerated hydratation and faster geopolymerization. Samples cured at higher temperatures shows the increased porosity in comparison to ambient cured samples [4]. Altan et al. suggested that when alkali activated slag mortars, using alkali hydroxide and a sodium silicate activator, were cured at room temperature (23 °C) for a sufficiently long time (70 days), an equal or higher strength could be attained compared to those cured at 80 °C for only 4 days [23]. Fernandez-Jimenez et al. reported the effects of curing conditions on alkali activated slag and fly ash based materials. Trends were reported whereby the compressive strength of activated fly ash with 8 M NaOH increased in a near linear fashion with both elevated temperatures (45, 65 and 85 °C) and curing time (5, 12, 24 h and 7 days). On the contrary, slag mortars activated with 4 M NaOH were reported to have a lower compressive strength after curing at an elevated temperature (45 °C) in comparison to those cured at room temperature. The difference was ascribed to the fact that the solubility of Al and Si increases significantly with temperature, but this is not so with the calcium compounds which were present in the slag [25]. Ozturk et al. investigated the alkali activation of electric arc furnace slag, where different silicate modulus, sodium concentrations, curing conditions and their effect on mechanical and microstructural behaviors were studied. They employed two curing temperatures (40, 80 °C) and two curing times (6, 12 h) both at two different percentages of relative humidity (45%, 98%). It was shown that the curing at higher relative humidity has beneficial effect on mechanical strength as less cracks appear in the moist conditions. Authors concluded that when the curing temperature is 40 °C and time of curing is prolonged from 6 to 12 h, the cracks occur due to evaporation of water and when the temperature is higher (80 °C), the hydratation reactions are faster resulting in the denser microstructure and therefore the mechanical strengths are increased. Also the higher amounts of silica content propagate the C-S-H gel formation which densify microstructure and therefore is one of the important factor in the strength development [5].

Although significant research has been done in the field of curing, and the usability of specific approaches for AAM systems has been confirmed, there is no comprehensive model for influential parameters in designing of optimal AAM mixtures available to date. Therefore in this case study, several mixtures with the Slag A/Slag R ratios 1/0, 3/1, 1/1, 1/3 and 0/1 and further activated with potassium silicate (with the activator/precursor ratio 1:2) were prepared. To optimal mixture, where Slag A/Slag R ratio was 1/1, different curing regimes were implemented in order to determine their influence on the development of mechanical strength. Curing was set at room temperature (R.T.), and in a heat-chamber at 50, 70 and 90 °C, and the effects of curing time for 1, 3, 7 and 28 days ware furthermore studied. Input materials as well as samples cured under different conditions were further microstructurally evaluated by means of XRD, FTIR, SEM and mercury intrusion porosimetry (MIP). 

## 2. Experimental Methods

### 2.1. Characterization of Materials

Two different types of metallurgical slag, electric arc slag (Slag A) and ladle slag (Slag R), from Slovenian metallurgical steel and iron plants were tested as potential precursors for alkali activation to be used in this study. Since it was received in aggregate form, milling and sieving into powder form of less than 63 µm was performed prior to chemical analysis, analysis of mineralogical composition and alkali activation. Elemental chemical composition was determined by X-ray fluorescence (XRF) with a Wavelength Dispersive X-ray Fluorescence (WD XRF) instrument (Thermo Scientific, Thermo electron SA, Ecublens, Switzerland). Samples were prepared by mixing slag with fluxana (lithium tetraborate and lithium metaborat 50%) at a ratio of 1:10, which was then melted at 1025 °C. Loss on ignition of the samples was determined according to EN 196-2:2013 (https://www.zag.si/ajax/DownloadHandler.php?file=1584, accessed on 5 April 2019). 

Determination of the mineral composition was performed using an X-ray powder diffractometer (Malvern PANalytical Empyrean, Surrey, United Kingdom) with CuKα radiation of λ = 1.54 Å. Intensity was scanned in the 2θ range from 4° to 70°, at a rate of 0.026°/min. Data was further analysed with X’Pert High Score Plus diffraction software from PANalytical (Malvern PANalytical Empyrean, Surrey, United Kingdom), using database PDF 4+2015 RDB powder diffraction files from the Inorganic Structure Database (ICSD). X-Ray analysis of alumina oxide powder as a standard reference material (NIST 676a) was performed for quantitative analysis control of multiphase mixtures and for Rietveld refinement of the powder X-ray diffractograms. Rietveld refinement was performed by X’Pert High Score Plus diffraction software with goodness of fit of 4.9.

Particle size distribution was measured using a CILAS 920 (Cilas, Orleans Cedex, France) particle size analyser. Milled and sieved (63 µm) precursor powder was dispersed with microscan dispersant type C (Quantachrome Corporation, Florida, USA) during the measurements. The specific surface area of precursors (BET) was determined by nitrogen adsorption at 77° K over a relative pressure range of 0.05–0.3 (Micromeritics ASAP 2020, Micromeritics, Norcross, GA, USA). Prior to BET analysis samples were heated at 70 °C for 2 h and degassed to 10^−3^ Torr (Micromeritics Flowprep equipment, Micromeritics, Norcross, GA, USA).

The bending and compressive strength of several AAM samples was firstly measured after 3 days of curing at 70 °C. After selecting the optimal mixture, the mechanical strength of the AAM samples was measured after treatment in a heat-chamber at room temperature and at elevated temperatures (R.T., 50, 70, and 90 °C), for 1, 3, 7 and 28 days, using a Toninorm instrument (ToniNORM, ToniTechnik, Berlin, Germany) at a force rate of 0.05 kN/s. Bending and compressive strength was measured on 4 sample prisms (20 × 20 × 80 mm^3^).

The microstructure of the AAM samples was investigated by scanning electron microscopy (JSM-5500LV, Jeol, Tokyo, Japan) and energy dispersive spectroscopy (EDX, Link Pentafet, Oxford Instruments), using back-scattered electrons (BSE, SEM, JSM-5500LV, Jeol, Tokyo, Japan) in low vacuum (JSM-5500LV, Jeol, Tokyo, Japan). Prior SEM scanning the samples were vaccum-dried and sputter-coated with gold. 

The degree of the reaction was analysed using Fourier-transform infrared spectroscopy (FTIR, Perkin Elmer Spectrum two, Kentucky, USA), a technique used to obtain an infrared spectrum of AAM solid products [26]. 

Following the processes of curing and drying, mercury intrusion porosimetry (MIP) was performed on AAM samples using a mercury porosimeter (Micromeritics, Norcross, GA, USA). Results of porosimetry are determined as a function of mercury, which penetrates into porous samples under high pressure. 

### 2.2. Sample Preparation

Alkali activation was performed on both the A and R slags using a potassium silicate (K_2_SiO_3_) based binder Betol K 5020 T (Woellner, Germany). The particle size distributions as cumulative values at 10, 50 and 90% (C 10, C 50, C 90), and the specific surface area of slags A and R are presented in Table 1. Specifications of K 5020 T potassium glass are as follows: pH is 12.5, solid content is 48.5%, and mass ratio of K_2_O to SiO_2_ is 0.62 (K_2_O = 18.46%, SiO_2_ = 30.00%).

Various mixtures were prepared in ratios presented in Table 2 (A/R: 1/0, 3/1, 1/1, 1/3 and 0/1 and activator/slag ratio 1:2). In order to study the influence of curing on the mechanical strength of the specimens, prisms of the selected optimal mixture (A/R: 1/1) were exposed to different temperatures (Room Temperature (R.T.), 50, 70, and 90 °C) in a heat-chamber for 1, 3, 7 and 28 days.

## 3. Results and Discussion

### 3.1. Analysis of Raw Materials

Chemical analysis (XRF) of both types of slag is presented in Table 3. Slag A is an electric arc furnace type slag, while Slag R is a ladle furnace basic slag produced in the secondary stage of refining, when the steel is desulfurized. Due to these differences in production, the content of elements varies; for example Slag A has a higher content of SiO_2_ and Al_2_O_3_ than Slag R, but a lower content of CaO and MgO. 

**Figure 1:**
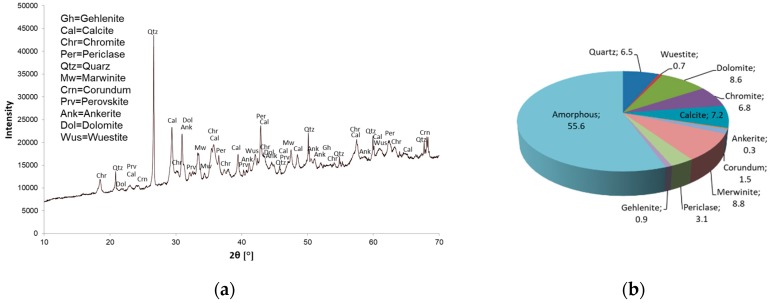
Powder X-Ray diffractogram of Slag A (**a**) and Rietveld quantification (all in %) of the phases in Slag A (**b**).

**Figure 2:**
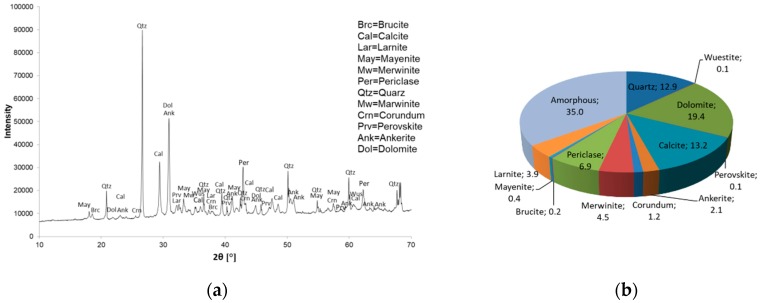
Powder X-Ray diffractogram of Slag R (**a**) and Rietveld quantification (all in %) of the phases in Slag R (**b**).

The mineral composition and quantitative determination of mineral phases (XRD and Rietveld) are shown in Figure 1 for Slag A and in Figure 2 for Slag R. Powder X-ray diffraction analysis confirmed the presence of amorphous phase in both A and R precursors. Crystals of quartz, calcite, dolomite, merwinite, periclase, ankerite and wuestite were present in both precursors. The Al to Si ratio was 1:1.66 for Slag A and 1:0.23 for Slag R. Rietveld analysis revealed differences in the quantity of amorphous and crystalline phases from the X-ray precursor powder diffractograms. In the case of precursor A the amount of amorphous phase was over 55%, whereas precursor R contained 35% amorphous phase. 

To find optimal strength (both bending and compressive), the investigation first focused on the influence of different Slag A/Slag R ratios as follows: 1/0, 3/1, 1/1, 1/3 and 0/1 with fixed activator to slag ratio 1:2. The precursor ratio was determined on the basis of XRF and XRD results. The mixture ratio was further calculated according to the theoretically ideal ratios between Na, K, Al and Si (Table 2) [27]. Due to a small liquid content in the calculated mixtures, a larger proportion of potassium glass and distilled water (4.5 wt % overall) were added in order to produce homogenous pastes. 

### 3.2. Analysis of AAM

#### 3.2.1. Influence of the Slag A/Slag R Ratio on Mechanical Properties

**Figure 3:**
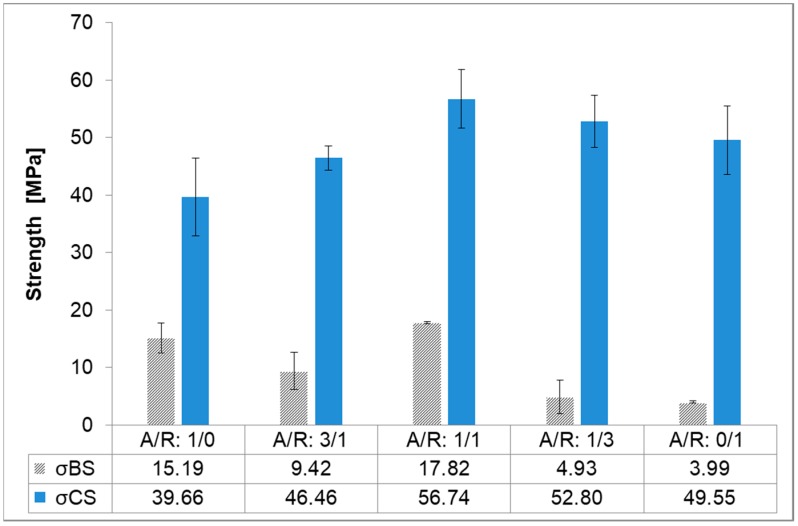
Bending (σBS) and compressive strengths (σCS) for AA mixtures of A and R slag.

Based on the results of the mechanical properties, as presented in Figure 3, both the maximum bending (σBS) and compressive strength (σCS) was achieved in the case of the mixture AR 1/1, in which bending and compressive strength were measured as 17.8 MPa +/− 1.2 MPa and 56.7 MPa +/− 2.3 MPa, respectively. In other mixtures σBS ranged from 5 to 17 MPa and σCS from 39 to 56 MPa. According to the (Na + K)/Al/Si ratio, as well as the measures of strength, the mixture with a ratio of A/R: 1/1 was confirmed to be the optimal mixture and therefore selected for further study on the effects of curing on the development of mechanical strength. 

#### 3.2.2. The Influence of Curing Temperature and Time on Mechanical Properties

**Figure 4:**
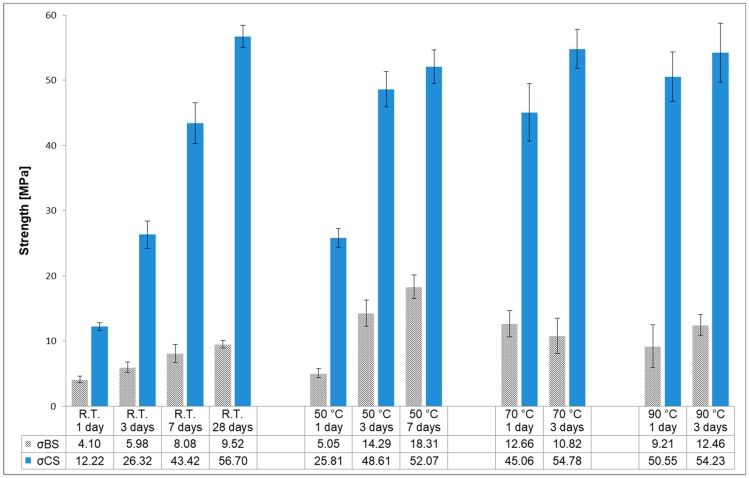
Bending (σBS) and compressive strengths (σCS) of A/R: 1/1 specimen cured at different temperatures (room temperature (R.T.), 50, 70 and 90 °C) and cured for different period of time.

Because the mechanical properties of AAM are also affected by curing temperature and curing time, bending and compressive strength were evaluated after 1, 3, 7 and 28 days for specimens cured under ambient conditions, after 1, 3, and 7 days for specimens exposed to 50 °C and after 1 and 3 days for specimens cured at 70 and 90 °C. The results are shown in Figure 4. The reaction of alkaline activation is increased at an elevated temperature during the early stages of curing. It has a beneficial effect on mechanical strength during the initial stage and it is known as accelerated curing [25]. Nevertheless, specimens cured for 28 days at room temperature also developed comparable bending and compressive strength to those specimens exposed to elevated temperatures. In that case, in order to achieve acceptable values of mechanical strength at lower temperatures, a longer curing time is needed [23]. The maximum value of bending strength for specimens treated at 50 °C was 18MPa +/− 1 MPa after 7 days. Values of compressive strength are comparable among all specimens; regardless of at which temperature they were cured, with greatest strength being developed after the longest exposure, attaining maximum values of approximately 56 MPa +/− 2 MPa. It is obvious that values of bending and compressive strength increase nearly linear with time for the samples cured at room temperature. The increase of compressive strength of sample cured at 90 °C for 1 day to 3 days is approximately 8%. Similar increase is found in sample exposed to 70 °C for 1 to 3 days. This suggests that the alkali activation is accelerated within the first day of curing where sample gains about 92% of all compressive strength. Elevated curing temperature above 70 °C has greater impact to the compressive strength development than curing time. The reason for this behavior could be that temperature accelerates the solubility of Al and Si which increases significantly with temperature [4,25]. The trend of bending strength for samples cured at elevated temperatures at 70 or 90 °C was not as consistent as for compressive strength, where the slight decrease was seen, respectively [28]. The variation of bending strength for those samples could be due to faster drying/hydratation/gel formation which leads to chemical deformation (expansion and shrinkage) resulting in self-induced stress [29]. 

#### 3.2.3. FTIR Evaluation 

In order to ascertain differences between the infrared spectrum of raw materials both precursors were first analysed by FTIR. Solid AAM products (cured at different temperatures) were then scanned by FTIR in order to follow the reaction over time.

**Figure 5:**
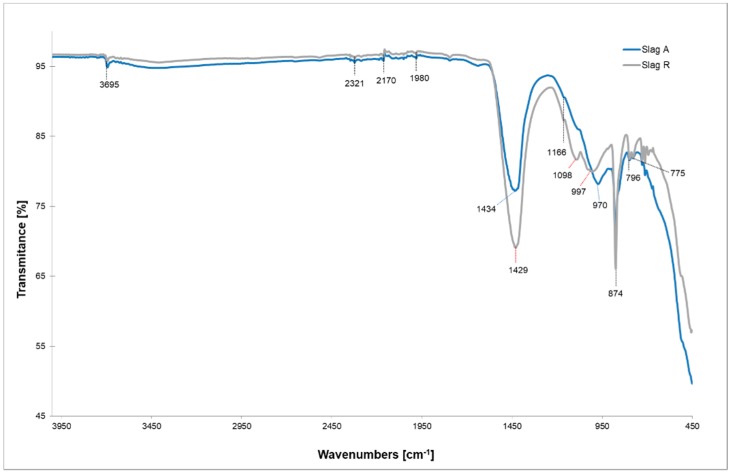
FTIR spectra of precursor materials (Slag A and Slag R).

**Figure 6:**
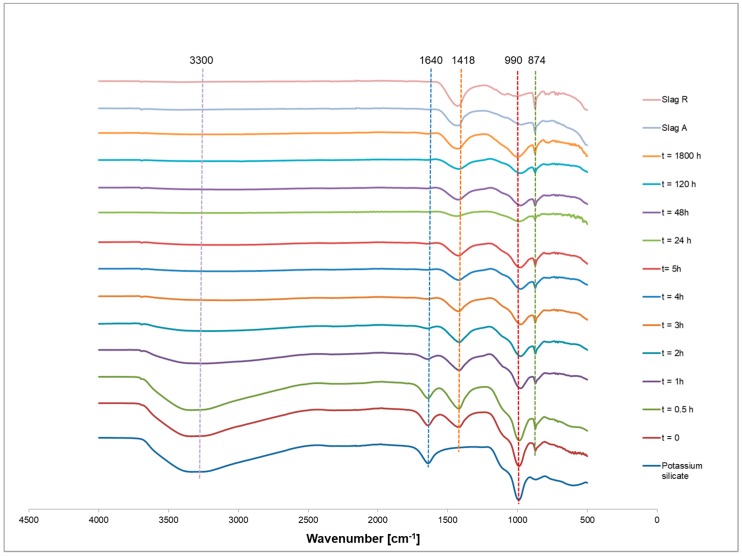
Time dependent FTIR spectra of for the precursors, alkali activator and AAM (A/R: 1/1 specimen cured at room temperature).

Figure 5 shows the IR spectrum of both unreacted slags. The major band lies in the 970–1160 cm^−1^ range, corresponding to the asymmetric stretching vibration of Si–O–Si and the Si–O–Al bonds [30]. The intensive bands at 1434 and 1429 cm^−1^ (for Slag A and Slag R, respectively) are related to the modes of CO_3_ contained in CaCO_3_, as also confirmed by XRD analysis. It has been reported that this main carbonation band also has a peak at 874 cm^−1^, which is also seen in both slags as a very intensive and sharp peak [31]. Due to the unreactive nature of this crystalline phase it stays present also after the alkali activation process (Figure 6). The peak that indicates the presence of quartz is observed at 796 cm^−1^ [32]. 

Figure 6 shows the time-dependent changes in the IR spectra of potassium silicate-activated slag pastes (A/R: 1/1) for t = 0, 0.5, 1, 2, 3, 4, 5, 24, 48, 120 and 1800 h, as well as the IR spectra of both slags and potassium silicate. Signals attributed to the O–H symmetric stretching and H–O–H bending vibrations in activated materials appear at around 3300 and 1640 cm^−1^ respectively and then decrease over time due to a lower concentration of available water as it is consumed into products of the reaction [33]. 

**Figure 7:**
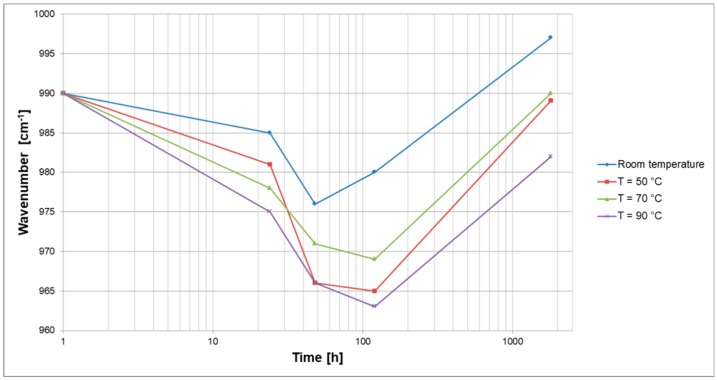
Si-O-T (T = Si or Al) band shifts of activated slag pastes A/R: 1/1 cured at different temperatures.

Peaks at approximately 1418 cm^−1^ and 990 cm^−1^ correspond to the Si–O vibrations from potassium silicate, and also reduce as the length of the process increases. The major band appears in the 900–1100 range, assigned to the vibration of Si–O–T (T = Si or Al) bonds. A typical representation of changes in wavenumbers attributed to the Si–O–T band shift is shown in Figure 7. In all cases, there is firstly a drop in the peak position of a wavenumber, followed by a gradual increase, which could be attributed to the severing of pure silica bonds and incorporation of Al into the gel [34,35], an effect which becomes more pronounced at a higher curing temperature.

#### 3.2.4. Microstructural Evaluation

**Figure 8:**
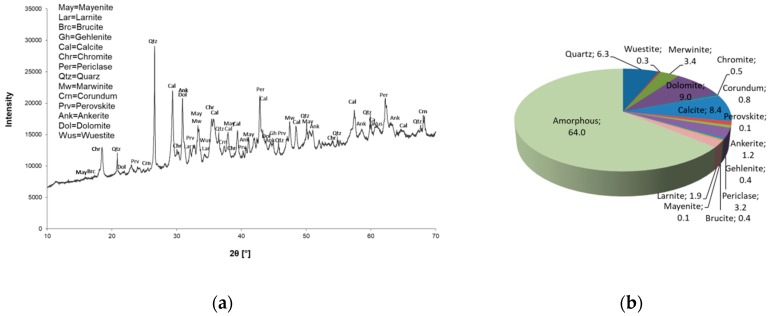
Powder X-Ray diffractogram of A/R: 1/1 specimen cured at 70 °C for 3 days (**a**) and Rietveld quantification (all in %) of the same specimen (**b**).

The powder X-ray diffractogram and Rietveld quantitative analysis of AAM are presented in Figure 8. Crystal patterns of AAM show the presence of all minerals obtained from each precursor, but in a different quantity from their original states. Determination of mineral phases by means of results from Rietveld analysis shows quantitative differences prior to and after alkali activation. The quantity of amorphous phase determined from Rietveld is around 64% in specimen A/R: 1/1 (Figure 8), whilst the arithmetic value calculated from a ratio of Slag A/Slag R (in ratio 1/1), based on the amount of amorphous phase in the precursors (Figure 2b and Figure 3b), would be approximately 40%. The Rietveld technique is employed as a very precise method [36], so the increase in amorphous phase is partially ascribed to added water glass (solid content is 48.5%), and additionally to the highly amorphous calcium silicate hydrate gel C–S–H, which is formed as part of the reaction between the activator and Ca^2+^ from the precursor [35,37]. The quantity of mineral phases after alkali activation and treatment are found to be present in similar quantities as prior to activation, except calcite, which decreased more significantly. 

**Figure 9:**
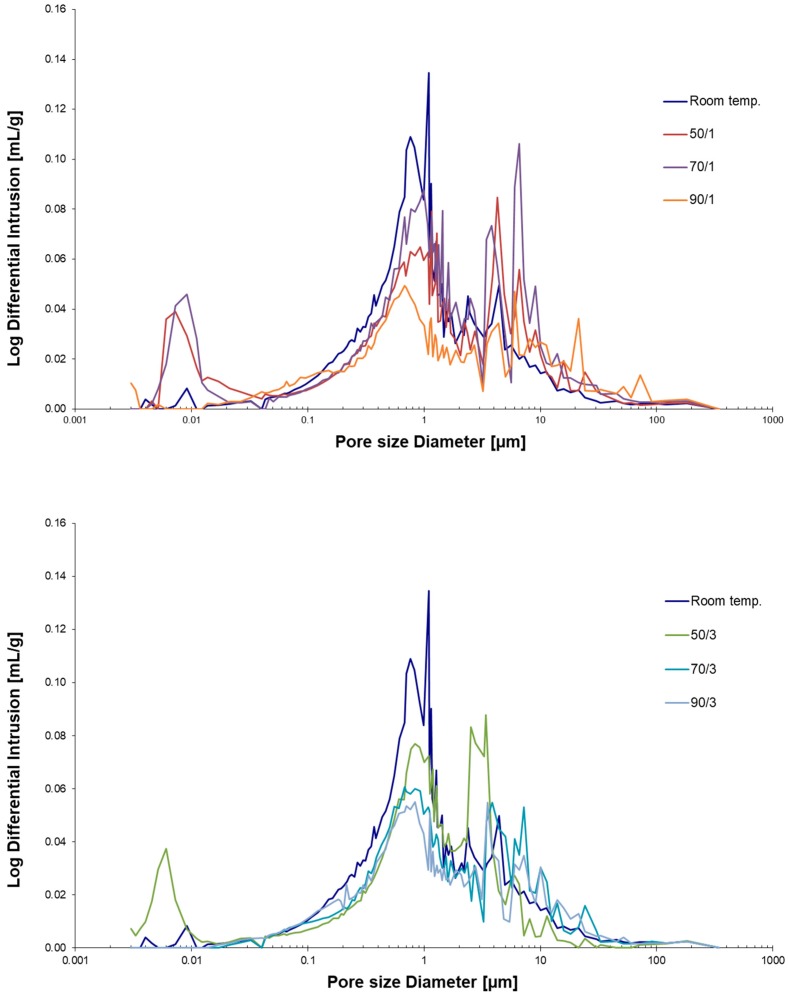
Differential mercury intrusion porosimetry of A/R: 1/1 specimen cured at different temperatures (R.T., 50, 70, 90 °C) for one (**above**) or three days (**bottom**).

Analysis of porosity made by mercury intrusion (MIP) is presented in Figure 9. The lowest porosity was found in A/R: 1/1 cured at 90 °C for 3 days, with a value of 15.41%. Samples cured at room temperature and measured after 28 days had a porosity of 19.51%, and the highest peak of pore distribution was around 1 µm. Samples cured at elevated temperatures showed bimodal pore distributions with maximum peaks at 0.5 µm and around 10 µm. Porosity decreases with curing temperature, a trend especially obvious after 3 days of curing, the main reason for which could be a faster evaporation of water (which is thus not being spent for the reaction). The decrease in porosity after curing is primarily attributed to a reduction in the overall proportion of pores with a diameter smaller than 1 µm, and it is worth noticing that pores around 0.01 µm practically disappeared when cured at 90 °C for 1 day and at 70 °C for 3 days. The porosity values of AAM samples, as measured by MIP, are presented in Table 4. The initial pores in AAM can occur when the paste is gravity-cast in the moulds [38], and/or after alkali activation, then in the early stages of the curing process more pores and micro-cracks are produced which contribute to drying shrinkage [39]. The volume of fine pores decreases if C-S-H gel is formed in the AA reaction, especially at elevated temperatures [40].

**Figure 10:**
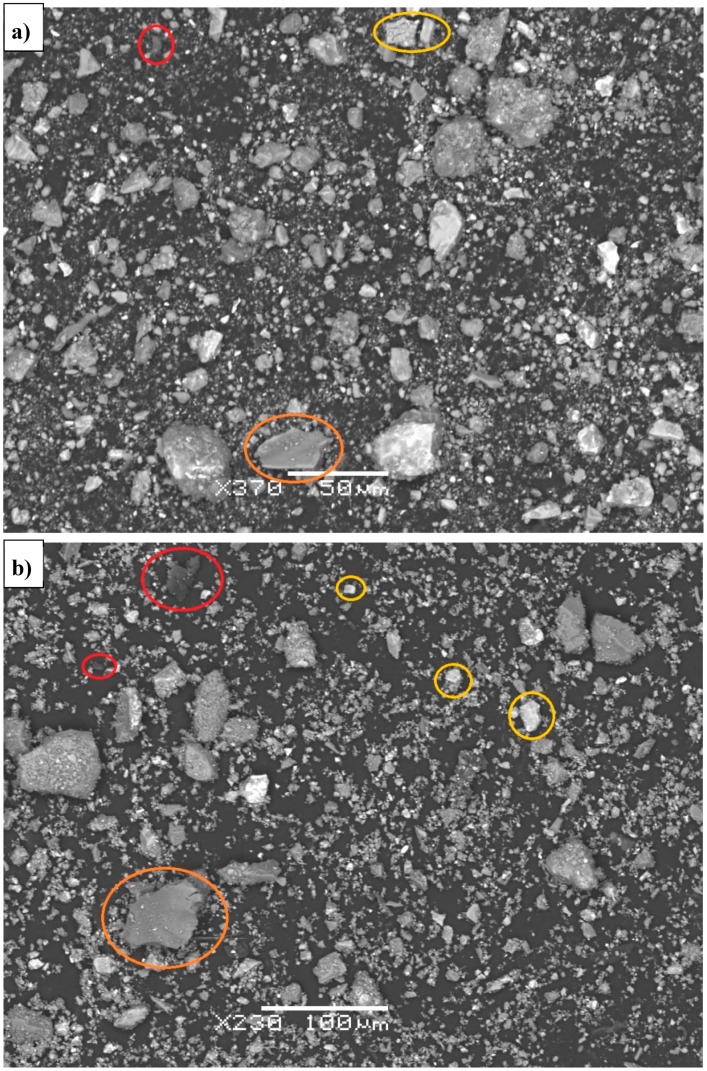
SEM scan of Slag A (**a**) and Slag R (**b**).

**Figure 11:**
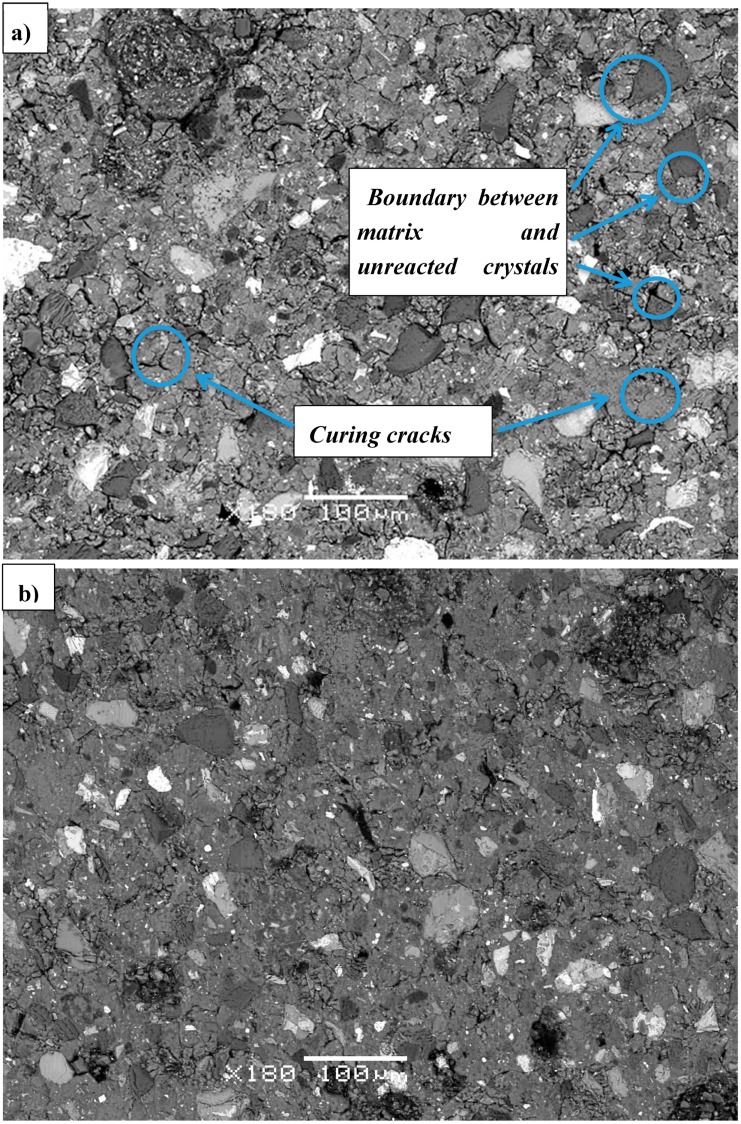

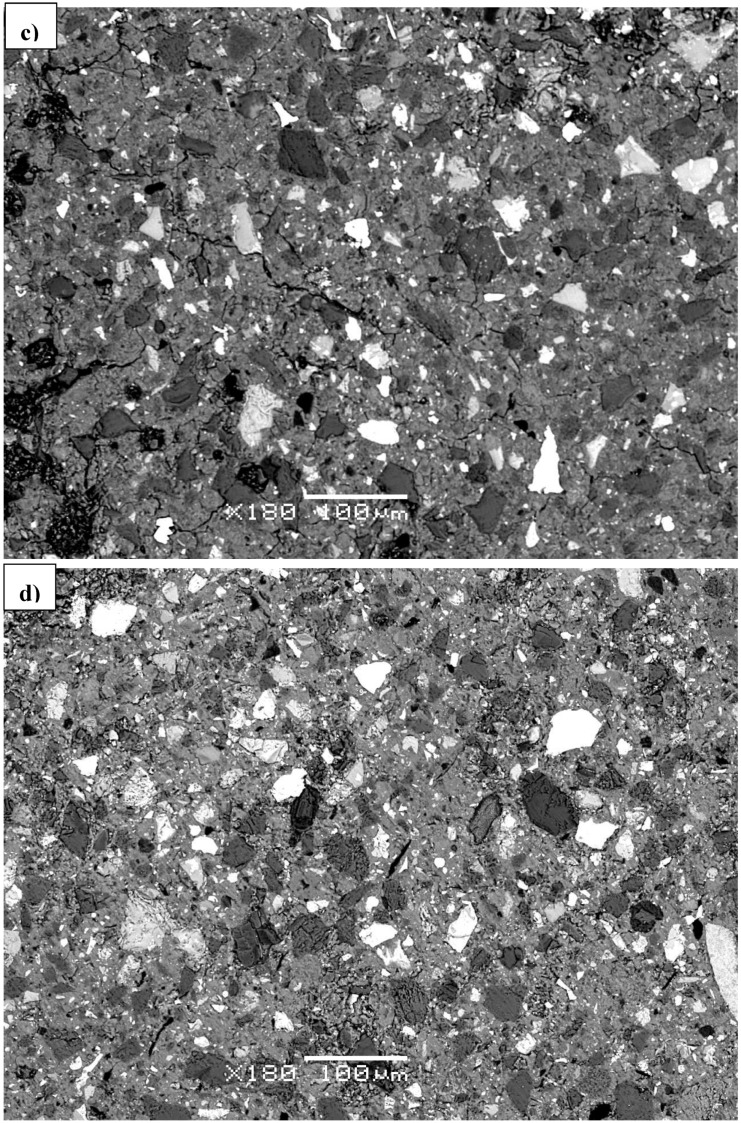
SEM images of A/R: 1/1 sample cured at: (**a**) R.T. (28 days) (**b**) 50 °C, 1 day, (**c**) 50 °C, 3 days, (**d**) 90 °C, 3 days.

**Figure 12:**
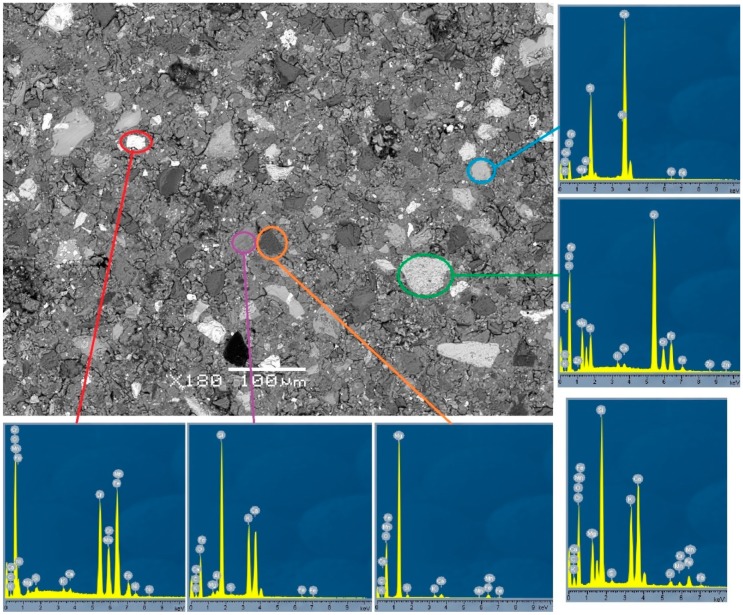
EDX analysis of A/R: 1/1 sample cured at 70 °C for 1 day. The unmarked EDX shows overall analysis of the AAM, purple is matrix, the rest present unreacted particles from slags.

Microstructural analysis was carried out by SEM investigation of the Slag A and Slag R powder (Figure 10a,b) and on the polished surface of A/R: 1/1 specimens treated at elevated temperatures for different curing times (Figure 11a–d). The main elements in AAM were determined with Energy Dispersive Spectroscopy (EDX), and are shown in Figure 12. From the scans of precursors (Figure 10a,b) it is obvious that distribution of the particles is not uniform and is comparable for both milled and sieved slags. The shape of particles is random with many sharp edges due to the milling. The different particles’ shades of gray are defining different elemental structure, where particles, according to chemical analysis performed by Energy Dispersive X-Ray Spectroscopy (EDX) presented on Figure 12, in red circles contain more Mg, particles in orange circles more Si, and particles in yellow circles more Ca. There are many particles seen also after activation (Figure 12) that have similar elemental structure as particles in slags (Figure 10), meaning that they did not form during AA, since those particles did not react with alkali, and present in AAM only the filling. EDX of AAM (Figure 12) area circled with purple presents matrix, where element in abundance is Si, followed by K and Ca. On Figure 11a,b are marked different defects present in AAM.

The volume of cracks and pores is affected by the alkaline reaction and the curing conditions, where water is removed from the AAM matrix [4,5]. The distribution of particles within the matrix of all samples is similar for all samples, but some cracks and voids are visible in the sample cured at room temperature, especially around single grains (Figure 11a,b). In the sample cured at 50 °C, for 3 days, those cracks propagate through the matrix (Figure 11c), while at higher temperatures (70 °C and 90 °C) the microstructure is denser and the cracks are not present (Figure 11d). That could be due to accelerated reactions at elevated temperatures of curing or due to formation of C-S-H gel [4]. As expected, the mechanical strength values (as presented in Figure 4) increased with fewer cracks and lower porosity. 

## 4. Conclusions

Two types of metallurgical slag from Slovenia, electric arc furnace slag (Slag A) and ladle slag (Slag R), were investigated for potential use in synthesis of AAM. Slags were characterized and it was confirmed that both contain amorphous phase of Si and Al which is a base for alkali activation. In order to find the optimal mixture, preliminary alkali activation tests were employed with different Slag A/Slag R ratios (1/0, 0/1, 1/3, 1/1 and 3/1) and activated with potassium silicate using an activator to slag ratio of 1:2. The values of bending strength ranged from 4 to 18 MPa, whilst compressive strength varied from 40 to 58 MPa. 

The optimal mixture was found to be A/R: 1/1, which was further investigated to study strength development under the influence of different curing regimes at room temperature (R.T.), and in a heat-chamber at 50, 70 and 90 °C, and the effects of different curing times for 1, 3, 7 and 28 days. For samples cured at room temperature almost linear trends were shown between mechanical strength and both curing time and temperature. The bending and compressive strength of samples cured at 70 °C for 3 days and samples cured at R.T. for 28 days were almost identical, i.e., 56 ± 2 MPa. Higher curing temperatures resulted in increased compressive strengths. 

Microstructural analysis has shown that:

- with the in-situ FTIR measurements two phenomena are observed: (i) a decrease in the intensity of the H_2_O bands consumed by reaction and/or evaporation (at about 3300 cm^−1^ and 1639 cm^−1^) and (ii) a condensation reaction, evidenced by the displacement of the main peak around 980 cm^−1^, as the time and temperature of curing increased.
- MIP analysis showed a reduction in porosity when curing occurred at an elevated temperature, primarily due to a reduction in the proportion of pores with a diameter of less than 1 µm.
- SEM analysis confirmed the presence of micro cracks which are present in the samples cured under 70 °C for one day. Above this temperature and time exposure cracks are not detected any more.

It has been confirmed that curing regime is crucial factor affecting the mechanical as well as microstructural properties of slag based alkali activated materials.

## Figures and Tables

**Table 1:** The particle size distribution of used precursors.

Precursor	C 10 (µm)	C 50 (µm)	C 90 (µm)	Surface Area—BET (m^2^/g)
Slag A	0.80	5.94	28.71	7.61
Slag R	0.83	5.45	24.75	3.52

**Table 2:** Overview of the composition of different mixtures prepared for the investigation with the calculated (Na+K)/Al/Si ratios in the precursor material as well as prepared mixtures.

Sample Designation	Slag A [wt %]	Slag R [wt %]	K_2_SiO_3_ [wt %]	Ratio of (Na+K)/Al/Si in Precursors	Ratio of (Na+K)/Al/Si in Prepared Mixture
A/R: 1/0	66.6	/	33.3	0.08:1:1	1.4:1:3.33
A/R: 3/1	49.9	16.7	33.3	0.09:1:1.4	1.52:1:3.21
A/R: 1/1	33.3	33.3	33.3	0.11:1:1.09	1.67:1:3.07
A/R: 1/3	16.7	49.9	33.3	0.13:1:0.72	1.84:1:2.9
A/R: 0/1	/	66.6	33.3	0.15:1:0.23	2.05:1:2.69

**Table 3:** The chemical composition of Slag A and Slag R used in this study.

Elements (wt %)	SiO_2_	Al_2_O_3_	Fe_2_O_3_	CaO	MgO	Na_2_O	K_2_O	Cr_2_O_3_	MnO	LOI	OTH
Slag A	21.05	8.54	11.37	20.87	14.87	0.13	0.17	3.76	2.24	14.15	2.80
Slag R	13.69	5.20	4.64	27.85	23.25	0.28	0.14	0.18	0.62	20.47	4.43

**Table 4:** The values of differential MIP porosimetry.

	R.T.	50/1	50/3	70/1	70/3	90/1	90/3
**Total pore area [m^2^/g]**	2.12	2.06	2.11	2.03	2.61	2.19	2.17
**Porosity [%]**	19.51	20.10	18.71	22.92	17.02	15.88	15.41
**Median Pore Diameter (Volume) [µm]**	0.91	1.02	1.02	1.20	1.33	1.31	1.13
**Median Pore Diameter (Area) [µm]**	0.017	0.008	0.006	0.008	0.151	0.004	0.113
**Average Pore Diameter (4V/A) [µm]**	0.23	0.05	0.04	0.07	0.50	0.14	0.39
**Apparent (skeletal) Density [g/mL]**	2.63	2.59	2.59	2.63	2.61	2.60	2.57
